# Validation of human papillomavirus genotyping by signature DNA sequence analysis

**DOI:** 10.1186/1472-6890-9-3

**Published:** 2009-05-22

**Authors:** Sin Hang Lee, Veronica S Vigliotti, Jessica S Vigliotti, Suri Pappu

**Affiliations:** 1 Department of Pathology, Milford Hospital, Milford, Connecticut, USA

## Abstract

**Background:**

Screening with combined cytologic and HPV testing has led to the highest number of excessive colposcopic referrals due to high false positive rates of the current HPV testing in the USA. How best to capitalize on the enhanced sensitivity of HPV DNA testing while minimizing false-positive results from its lower specificity is an important task for the clinical pathologists.

**Methods:**

The HPV L1 gene DNA in liquid-based Pap cytology specimens was initially amplified by the degenerate MY09/MY11 PCR primers and then re-amplified by the nested GP5+/GP6+ primers, or the heminested GP6/MY11, heminested GP5/MY09 primers or their modified equivalent without sample purification or DNA extraction. The nested PCR products were used for direct automated DNA sequencing. A 34- to 50-base sequence including the GP5+ priming site was selected as the signature sequence for routine genotyping by online BLAST sequence alignment algorithms.

**Results:**

Of 3,222 specimens, 352 were found to contain HPV DNA, with 92% of the positive samples infected by only 1 of the 35 HPV genotypes detected and 8% by more than 1 HPV genotype. The most common genotype was HPV-16 (68 isolates), followed by HPV-52 (25 isolates). More than half (53.7%) of the total number of HPV isolates relied on a nested PCR for detection although the majority of HPV-16, -18, -31, -33 -35 and -58 isolates were detected by a single MY09/MY11 PCR. Alignment of a 34-base sequence downstream of the GP5+ site failed to distinguish some isolates of HPV-16, -31 and -33. Novel variants of HPV with less than "100% identities" signature sequence match with those stored in the Genbank database were also detected by signature DNA sequencing in this rural and suburban population of the United States.

**Conclusion:**

Laboratory staff must be familiar with the limitations of the consensus PCR primers, the locations of the signature sequence in the L1 gene for some HPV genotypes, and HPV genotype sequence variants in order to perform accurate HPV genotyping.

## Background

In the United States, approximately 20 million people are currently infected with human papillomavirus (HPV) [1], and 11,070 women are expected to be diagnosed with cervical cancer in 2008 [2]. To further reduce the cancer risk, testing high-risk HPV DNA in liquid-based cervicovaginal Pap cytology specimens has been recommended in the practice guidelines to direct clinical management of individual patients with a cytologic classification of atypical squamous cells of undetermined significance (ASCUS) [3-5]. Using the histological grade of CIN3/2 in the subsequent colposcopic biopsy as the end point for evaluation, the sensitivity of the commercially available high-risk hc2 HPV assay (Digene, Gaithersburg, MD) is reported to be 50–75% [6,7], and its specificity 15–71% [6,8,9], depending on the age of the patients, the patient population and the Pap cytology used in combination for triage. A recent comprehensive analysis has confirmed that screening with combined cytologic and HPV testing, regardless of patient age, leads to the highest number of excessive colposcopic referrals due to high false positive rates of the current HPV testing. According to this analysis, more than 95% of referrals to colposcopy for diagnostic workup are false positive and/or potentially excessive. How best to capitalize on the enhanced sensitivity of HPV DNA testing while minimizing false-positive results from its lower specificity is an important question to be addressed in upcoming screening guidelines [10].

In part due to the lack of a commercial type-specific HPV test, about 19% of the clinical laboratories surveyed in 2006 by the College of American Pathologists (CAP) relied on using a laboratory-developed test for HPV assay [11]. The CAP has advised that rigorous validation is required for offering a non-FDA-approved HPV test in certified high-complexity testing laboratories under CLIA 88 [12,13]. A 92% CIN 3 detection rate has been proposed as the clinical sensitivity along with an 85% CIN 3 confirmation as the clinical specificity for validation of all HPV assays [14]. However, the results were largely disappointing when attempts were made to use a yardstick based on interpretative human observations, such as the CIN grading, to validate the sensitivity and specificity of a molecular virology test [6-9] which is governed by the law of physics, or vice versa [15].

Significant differences were noted in the detection of HPV infection and individual genotypes when different probe hybridization methods were used to test the same group of specimens [16]. The SPF10-line probe assay (LiPA; DDL Diagnostic Laboratory; Voorburg/The Netherlands) detected more HPV-31 than HPV-16 while the line blot assay (LBA; Roche Molecular Systems, Pleasanton, CA) detected more HPV-16 than HPV-31 in the same samples, as reported by two independent groups of authors [16,17]. In addition, the LBA detected significantly more HPV-42, HPV-56 and HPV-59 whereas the LiPA detected more HPV-52 when these two hybridization methods were compared in parallel studies [17]. These discrepancies in HPV genotypes found in the same group of samples suggest that some HPV variants might have been identified as different genotypes by different probe hybridization methods. The US Food and Drug Administration (FDA) has concluded "*Complex probe cocktails may crossreact and/or compete with one another. There is currently no standardized clinical panel of representative HPV samples available against which specific probe combinations can be evaluated for clinical effectiveness." *[18]

By definition, a genotype of HPV differs in the L1 gene DNA sequence by at least 10% from every other known HPV type. HPV subtypes are those having DNA sequence similarities between 90% and 98% with a prototype in its L1 gene; HPV variants are those having a DNA sequence identity of over 98% of a prototype [19]. To sequence the entire L1 gene (ca. 1.6 kb) for genotype determination of the HPV DNA detected in each and every positive clinical specimen is cost-prohibitive. However, it is possible to analyze a hypervariable DNA segment of the L1 gene and compare its sequence with those stored in the GenBank database for genotyping [20-25]. Several institutes have sequenced a 150 bp PCR amplicon defined by the GP5+/GP6+ consensus primers for accurate genotyping [20,22,23]. For routine practice, a sequence of 34 bases downstream of the GP5 binding site has been proposed for accurate genotyping of all clinically relevant anogenital HPV variants [20]. This study was designed to further explore the possibility of using this method of HPV genotyping for routine application in a clinical laboratory to improve the specificity of the current HPV testing and its potential pitfalls.

## Methods

The purified full-length plasmid DNAs of HPV types -6B, -11, -16, and -18 purchased from American Type Culture Collection (ATCC) were used as HPV DNA standards. The nested PCR products generated of these standard genotypes by the primers described below were confirmed by direct automated DNA sequencing to be the L1 gene DNA of the expected HPV genotype. This preliminary experiment was to demonstrate that the methodology used in this study was capable of generating genotype-specific template suitable for DNA sequencing.

The purified HPV type-16 DNA was used as the routine positive control and molecular grade pure water instead of DNA extract was used as negative control for each PCR run. To avoid cross contamination, three separate rooms with no air re-circulation were dedicated to nucleic acid amplification tests. Two of the rooms were each equipped with a 32" PCR workstation (AirClean Systems, Raleigh, NC). All pre-amplification procedures were performed in PCR station I. All post-PCR procedures were carried out in PCR station II, including preparations for the nested PCR and sequencing reaction. Gel electrophoresis and DNA sequencing were performed in the third isolation room. No post-PCR materials or any items contaminated by amplicons, or equipment used in the post-PCR rooms were allowed to enter the pre-PCR working space. The laboratory is certified by the State of Connecticut Department of Public Health under the Clinical Laboratory Improvement Amendments of 1988 (CLIA) to perform human papillomavirus (HPV), *Chlamydia trachomatis *and *Neisseria gonorrhoeae *tests by polymerase chain reaction (PCR) and DNA sequencing, based on methods previously published [26-28].

Three thousand two hundred twenty-two (3,222) alcohol-preserved Cytyc or Surepath liquid-based Pap cytology specimens submitted by gynecologists in southern Connecticut for routine HPV testing were used for this study. The patients included 2,633 Milford residents representative of a population in the rural and suburban United States with a cervical cancer rate less than 6.8 per 100,000 women [29]. In the latter resident group, the HPV positive rate among the patients below 30 years old was previously found to be 36.1%, and that for those 30 years and older was 7.3% [30]. The HPV tests were ordered for the patients 30 years and older (up to 65) as adjunctive screening to routine Pap cytology and for the patients below age 30 who had a cytology diagnosis of ASCUS or more severe cytological changes. Publication of the laboratory data with blinded patient identities was approved by the Milford Hospital IRB.

For HPV detection and genotyping, the pellet derived from 1 mL of the ThinPrep (Cytyc) or 0.5 mL of the SurePath cell suspension was washed in reagent grade water, then in 1 mL buffer consisting of 50 mM Tris-HCl, 1 mM EDTA, 0.5% Tween 20, pH 8.1. The washed cell pellet was re-suspended and digested at 45–55°C overnight in 100 μL of 0.1 mg/mL proteinase K (Sigma Chemical Co., St. Louis, MO) dissolved in the same washing buffer. After denaturing the proteins in the cell digestate in a metal block heated to 95°C for 10 min and a final centrifugation of the digestate at 13,000 rpm for 5 min, the supernatant was used for PCR without further purification.

For primary PCR amplification, 1 μL of the crude digestate, 1 μL of 10 μmolar MY09 primer, 1 μL of 10 μmolar MY11 primer and 2 μL of water were added to a PCR tube containing 20 μL of LoTemp™ HiFi^® ^DNA polymerase ready-to-use mix (HiFi DNA Tech, LLC, Trumbull, CT) which contained all the components needed for low temperature PCR, including dNTPs, Mg^++^, buffer, HiFi^® ^DNA polymerases, proprietary dsDNA melting agents and dNTP preservatives, to reach a final 25 μL reaction volume. For thermocycling, the temperature steps of a TC-412 Thermal Cycler (Techne Incorporated, Burlington, NJ) were programmed for an initial heating at 85°C for 10 min, followed by 30 cycles at 85°C for 30 sec, 40°C for 30 sec, and 65°C for 1 min. The final extension was 65°C for 10 min as previously described [26-28].

For the nested PCR, a "trace" of the MY09/MY11 PCR products was transferred by a glass rod of about 1.5 mm in diameter with clean wettable surface to a second PCR tube containing 25 μL of complete nested PCR reaction mixture consisting of 20 μL of LoTemp™ HiFi^® ^DNA polymerase ready-to-use mix, 1 μL of 10 μmolar GP5+ primer, 1 μL of 10 μmolar GP6+ primer and 3 μL of water, using the same thermocycling program described above. Alternatively, the GP5+/GP6+ nested primers were replaced by the GP6/MY11 heminested PCR primer pair or the optimized modified equivalent, HiFi HPV nested PCR primer pair (catalog #3002, HiFi DNA Tech, Trumbull, CT) to generate a 190–200 bp nested PCR product for improved base resolution in direct automated DNA sequencing.

After completion of the primary and the nested PCR, a 5 μL aliquot of the PCR products was pipetted out from each tube and mixed with 2 μL loading fluid for electrophoresis in a 2% agarose gel containing ethidium bromide. The gel was examined under UV light. Visualization of a 450 bp PCR product band in the MY09/MY11 lane and/or a 150 bp (190–200 bp when the GP6/MY11 heminested PCR or the HiFi nested PCR primers were used) band in the nested PCR lane provided presumptive evidence of an HPV DNA in the sample, pending genotyping by direct DNA sequencing for final validation.

When the aforementioned nested PCR failed to re-amplify an MY09/MY11 PCR amplicon to generate an expected 150–200 bp amplicon, a trace of the MY09/MY11 PCR products was subjected to a long nested PCR using the heminested GP5/MY09 primers or an equivalent optimized long nested PCR primer pair (catalog #3003) to generate a 380–395 bp amplicon for DNA sequencing. This 380–395 bp nested PCR amplicon is referred to as a "long nest" in this article, to be distinguished from the aforementioned 190–200 bp nested PCR products (short nest).

The positive nested PCR products were subjected to direct automated DNA sequencing without further purification. Briefly, a trace of the nested PCR products was transferred from the PCR tube with a calibrated glass rod described above into a reaction mixture containing 1 μL of GP6 (GP5 for long nest PCR amplicon) or the optimized HiFi HPV sequencing primer (catalog #6007), 1 μL of BigDye Terminator (v 1.1/Sequencing Standard Kit, Applied Biosystems, Foster City, CA), 3.5 μL 5× buffer, and 14.5 μL of water in a total volume of 20 μL. This reaction mixture was subjected to 20 enzymatic primer extension/termination reaction cycles, according to the protocol supplied by the manufacturer (Applied Biosystems). The final reaction mixture was loaded in an automated ABI 3130 four-capillary genetic analyzer for sequence analysis. Sequence alignments were performed against the standard HPV L1 gene sequences stored in the GenBank database by BLAST analysis for final validation of the HPV genotyping. An exclusive "100% identities" match between the query and subject sequences, returned by the on-line algorithm, was required for genotyping except for variants not yet recorded in the GenBank.

To distinguish the overlapping base-calling peaks on a computer-generated electropherogram which resulted from co-amplification of non-HPV DNA in the sample, from those which were caused by co-amplification of more than one HPV DNA, the agarose gel band containing the nested PCR amplicon was cut out and crushed in a 0.5 mL Eppendorf tube containing 10 μL water to elute the DNA in question. A trace of the eluted DNA was transferred into another nested PCR tube containing all the nested PCR components for a second nested PCR. After the low temperature thermocycling described above, a trace of the second nested PCR product was subjected to direct automated DNA sequencing, and its electropherogram was compared with that using the first nested PCR amplicon as the sequencing template.

One μL of each digestate sample was placed in a separate PCR tube with a β-globin primer pair for human genomic DNA amplification as a control of specimen adequacy. Specimens with no β-globin gene amplification were excluded as insufficient.

## Results

A total of 352 among the 3,222 specimens submitted for HPV detection and genotyping were found to be positive for HPV DNA, including 20 positive repeat tests. Since HPV testing was ordered for patients below age 30 only when there was a history of abnormal Pap cytology, the HPV positive rate of the specimens from this group was about 5 times higher than that in those collected from patients age 30 or older [30].

Of the 352 specimens tested positive for HPV DNA, 324 (92%) were found to be infected by a single HPV genotype and 28 (8.0%) by more than one HPV genotype. There were 35 single HPV genotypes identified, including HPV-6, -11, -16, -18, -31, -32, -33, -35, -39, -40, -45, -51, -52, -53, -54, -56, -58, -59, -61, -62, -66, -67, -68, -69, -70, -71, -72, -73, -74, -81, -83, -84, -86, -87, and -91, all validated by a segment of signature sequence within the L1 region flanked by the MY09 and MY11 PCR primers, and confirmed by the GenBank database. The efficiency of DNA amplification of the individual HPV genotypes by the chosen consensus PCR primers varied greatly and appeared to be type-dependent. While the majority of the positive nested PCR amplicons of HPV-16, HPV-18, HPV-31, HPV-33 and HPV-35 were associated with a well defined 450 bp band in the companion MY09/MY11 primary PCR gel, most of the other HPV genotypes depended on a nested PCR for detection (Table 1).

**Table 1:** HPV L1 gene amplification by MY09/MY11 PCR primers followed by positive short or long nested PCR

**HPV Type**	MY09/MY11+		MY09/MY11-		Total isolates
			
	Short	Long	Short_	Long	
			
**6**	8	0	12	N	20
**11**	1	0	0	N	1
**16**	40	0	28	N	68
**18**	16	0	5	N	21
**31**	11	0	4	N	15
**32**	3	0	0	N	3
**33**	4	0	1	N	5
**35**	6	1	0	N	7
**39**	4	1	7	N	12
**40**	2	0	3	N	5
**45**	4	0	5	N	9
**51**	2	0	0	N	2
**52**	9	0	16	N	25
**53**	2	1	0	N	3
**54**	5	0	13	N	18
**56**	4	0	11	N	15
**58**	6	0	3	N	9
**59**	4	0	8	N	12
**61**	2	0	5	N	7
**62**	2	0	5	N	7
**66**	0	0	9	N	9
**67**	0	0	1	N	1
**68**	0	0	1	N	1
**69**	0	0	2	N	2
**70**	1	0	5	N	6
**71**	0	0	2	N	2
**72**	4	0	2	N	6
**73**	4	0	7	N	11
**74**	0	0	1	N	1
**81**	3	0	4	N	7
**83**	2	2	1	N	5
**84**	2	0	1	N	3
**86**	0	0	4	N	4
**87**	0	0	1	N	1
**91**	0	0	1	N	1
Mixed	7	0	21	N	28
Total	158	5	189		352

MY09/MY11+ = 450 bp amplicon visualized on primary PCR gel.

MY09/MY11- = 450 bp amplicon not visualized on primary PCR gel.

Short = 150 bp or 190–200 bp nested PCR amplicon visualized.

Long = 380–395 bp nested PCR needed due to failure of short PCR.

N = not performed.

Underlined are "High-risk" HPVs targeted by Digene hc2 assay.

The GP5+/GP6+ nested PCR primers were effective, capable of generating a 150 bp nested PCR amplicon for most HPV isolates which had been previously amplified by the MY09/MY11 primers. In 189 (53.7%) of the specimens, a clearly defined target PCR product band was visualized in the nested PCR agarose gel, but not in the companion gel of the primary PCR amplified with the MY09/MY11 primers. The MY09/MY11 primary PCR products of 2 isolates of HPV-39, 1 isolate of HPV-59, 1 isolate of HPV-73 and 1 isolate of HPV-91 could not be amplified by the Gp5+/GP6+ nested PCR primers. These latter HPV isolates required a heminested PCR with the GP6/MY11 primers or the HiFi nested PCR primers (catalog #3002) to generate a 190–200 bp nested PCR amplicon from the primary PCR products for direct automated DNA sequencing.

**Figure 1:**
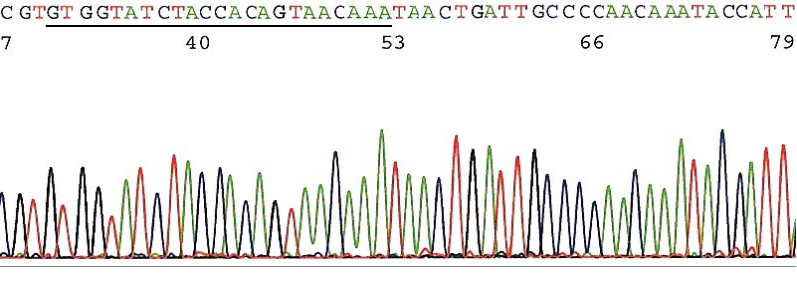
**A typical signature sequence excised from an electropherogram generated by direct automated DNA sequencing, including 23 bases of the GP5+ priming site (underlined)**. The template was generated by a pair of HiFi HPV nested PCR primers (equivalent to length flanked by GP6/MY11). A sequence of any 34 of these 53 bases confirms that this is an HPV-31 L1 gene DNA through BLAST sequence alignment algorithm. Any 50-base sequence in this region can be used for accurate genotyping of all known HPVs.

When the GP5+/GP6+ PCR primers were used to generate a 150 bp nested PCR amplicon for direct automated DNA sequencing and the GP6 nucleotide as the sequencing primer, the readily readable sequence for base calling on the electropherogram was 30 to 50 bases long immediately downstream of the GP5+ primer site. However, some variants of HPV-16, HPV-31 and HPV-33 share an identical 34-base sequence in this region (Table 2). The 190–200 bp amplicon generated by the GP6/MY11 heminested or the HiFi HPV nested PCR primers added the entire GP5+ primer site along with its adjacent upstream bases to the electropherogram for alignment algorithm. On this expanded electropherogram, any 34-base strand inclusive of the entire or part of the GP5+ primer site (Figure 1) could be used as the signature sequence to distinguish these 3 closely related "high risk" HPV genotypes. The exact location of the signature sequence in this hypervariable region may vary from genotype to genotype, based on the GenBank database. Any 50-base sequence from this region was found to be sufficient for validation of all HPV genotypes detected.

**Table 2:** HPV-16, -31 and -33 variants with close sequence similarity at the L1 gene GP5+ primer binding site

**Highly similar DNA sequence**	**Genotype**	**Locus**
5'_acgcagtacaaatatgtcattatgtgctgccata	HPV-16	EU779755
5'_acgcagtacaaatatgtcattatgtgctgccata	HPV-31	EF140820
5'_acgcagtacaaatatgtcattatgtgctgccata	HPV-33	DQ448214
5' tttgttggggtaaccaactatttgttactgttgt	HPV-16	EU779755
5' tttgttggggcaatcagttatttgttactgtggt	HPV-31	EU779751
5' tttgttggggcaatcaggtatttgttactgtggt	HPV-33	EU779744

DNA sequences retrieved from the National Center for Biotechnology Information database. The symbol _ and the underlined bases represent the end and the beginning of the GP5+ PCR priming site, respectively. A 34-base sequence shared by HPV-16 and certain HPV-31 (EF140820) and HPV-33 (DQ448214) strains is located immediately downstream of the GP5+ site. A 1- to 4-base difference is present upstream of the GP5+ site between certain variants of HPV-16, HPV-31 (EU779751) and HPV-33 (EU779744).

One isolate of HPV-39, 1 of HPV-35, 1 of HPV-53 and 2 of HPV-83, which were initially amplified by the MY09/MY11 PCR primer pair, were not re-amplified either with the GP5+/GP6+ or with the GP6/MY11 heminested or the HiFi nested PCR primer pair. But their MY09/MY11 amplicons were successfully amplified with the GP5/MY09 heminested or the HiFi "long nest" PCR primers (catalog #3003) to generate a 380–395 bp amplicon for DNA sequencing. According to information provided by the vendor (HiFi DNA Tech, LLC), the long nest PCR primers have been designed for amplification of the segment flanked by the GP5/MY09 primers.

**Figure 2:**
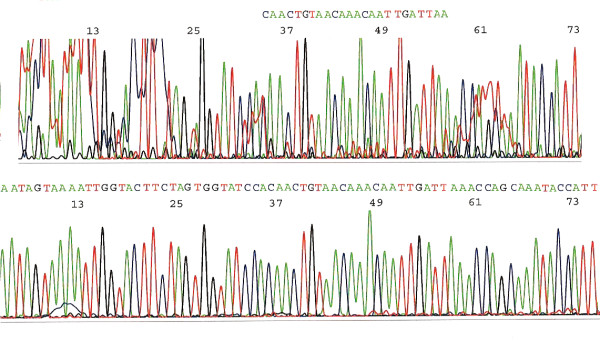
**A 30-year old patient (NB) had persistent positive "high-risk" HPV hc2 assays in spite of negative Pap cytology findings 3 years after a cone biopsy which confirmed a CIN3/2 lesion with HPV-52 DNA isolated from the archived paraffin-embedded tissue**. The electropherogram of DNA sequencing on a recent liquid-based cytology sample shows an ambiguous tracing (upper tracing). However, using a second nested PCR product as the template for sequencing reaction, the base-calling peaks (lower tracing) can be deciphered. Alignment of the complementary sequence in reading frame spaced as amino acid codons compared to nucleotides 6727–6798 of GenBank Locus AJ400628 is as follows: 5' aat ggt att tgc tgg ttt aat caa ttg ttt gtt aca gtt gtg gat acc act aga agt acc aat ttt act att Sample NB. 5' aat ggt att tgc tgg ttt aat cag ttg ttt gta acg gtt gtt gat act act cgc agt acc aat ttt act att AJ400628. Although the sequence of sample NB has a mismatch of 7 bases (underlined) against those of an HPV-87 prototype, the amino acid sequence, NGICWFNQLFVTVVDTTRSTNFTI, coded by both nucleotides is identical in this segment.

DNA sequencing of a short segment of the L1 gene for HPV genotyping was straightforward when a single HPV was present in the specimen. Overlapping and ambiguous base calling peaks on the computer-generated DNA sequencing electropherogram were caused by co-amplification of non-HPV DNAs by the consensus or degenerate PCR primers, or by co-amplification of more than one HPV genotypes in the sample. A double nested PCR procedure which re-amplified the first 190–200 bp nested PCR product excised from the agarose gel with a second nested PCR easily eliminated the non-HPV DNA amplicons which had interfered with the DNA sequencing process. In one case selected for illustration, this second nested PCR generated a more homogeneous template for DNA sequencing, resulting in a 72-base tracing with distinct base calling peaks (Figure 2) for BLAST analysis. The results of the sequence alignment algorithm suggested that this was a putative HPV-87 subtype having a "100% identities" match in amino acid sequence, but only 90% similarities in nucleotide sequence with the HPV-87 prototype stored in the GenBank database.

When a sample contained two or more genotypes of HPV DNA which were co-amplified during the first nested PCR and resulted in an ambiguous electropherogram with numerous overlapping base calling peaks, a second nested PCR could not generate a more homogeneous DNA template for DNA sequencing. For these mixed HPV infections, we relied on using specific HPV-16, -18, -6 and -11 DNA sequencing primers [31] to perform 4 individual DNA sequencing reactions to rule out the existence of these Gardasil^® ^vaccine-relevant HPVs [32].

## Discussion

The role of persistent infection by HPV as a tumor promoter in uterine cervical cancer induction has been recognized [33-37]. However, the issues of persistence of an HPV infection, the epidemiology of HPV infection, and the immunologic responses to an HPV infection are adequately studied only when HPV type-specific or perhaps even HPV variant-specific methods are available to the clinical laboratories which perform routine HPV tests for patient management. Since the FDA does not consider methods based on probe hybridization to be reliable for HPV genotyping [18], laboratory-developed HPV assays must be validated [12,13] by more stringent analyses.

Determination of the DNA base sequence of the L1 gene is the foundation of genotyping of all papillomaviruses [19]. However, DNA sequencing of the entire L1 gene [38] or performing multiple reactions to sequence a double-stranded template of the L1 gene from opposing ends [25] are beyond the financial feasibility of most clinical laboratories. A practical approach is to determine a short signature sequence in the hypervariable region of the L1 gene, which is unique for each HPV genotype as identified and confirmed by the GenBank database [20,22,23]. But, the cost for sample preparation and purification of the clinical materials prior to the DNA sequencing procedure is still inhibitory for implementation of such approach. The introduction of the low-temperature PCR system with a robust DNA polymerase, which does not require sample purification or DNA extraction, has simplified the process to direct DNA sequencing procedure, making rigorous validation of HPV genotyping in a clinical laboratory economically sustainable [26-28,30]. Using this system, the unpurified PCR products used for nested PCR and for enzymatic primer extension/termination reactions can be transferred by a small glass rod to avoid post-amplification micropipetting, which is the major source of cross contamination in routine diagnostic operations when a nested PCR is used.

We found that 189 of the 352 HPV isolates (53.7%) need a nested PCR for detection by agarose gel electrophoresis. The majority of HPV-16, HPV-18, HPV-31, HPV-33, HPV-35 and HPV-58 isolates are detected by the MY09/MY11 primary PCR alone (Table 1). But, 28 of the 68 (41.2%) HPV-16 isolates still depend on a nested PCR for detection. The HPV-18 DNA appears to be most effectively amplified since16 of the 21 isolates (76.2%) of HPV-18 were detected by a single MY09/MY11 PCR. Without a nested PCR, most of the samples containing HPV-39, HPV-52, HPV-54, HPV-56, HPV-59, HPV-66 and HPV-73 DNAs would have been classified as negative. Other authors have reported that a similar nested PCR amplification may increase the sensitivity of the single MY09/MY11 PCR by 10-fold [22]. Some even claimed that the sensitivity of a prototype PGMY09/11 single primer PCR assay is below that of the HPV hc2 assay although the hc2 assay might have classified some non-target "low-risk" HPVs as "high-risk" positives due to cross reactivity [39]. The nested PCR procedure described here detects 33.3% more sequencing-validated "high-risk" HPVs in liquid-based Pap cytology specimens than the hc2 assay when parallel tests were conducted on split samples [26], similar to the findings reported by others [22].

Our data have demonstrated that the sensitivity of the MY09/MY11 amplification of HPV DNA is relatively low. More than half of the positive cases are only detected in the nested PCR using GP6/MY11 primers after routine second amplification. In this protocol, only when a case was found to be positive for a 450 bp MY09/MY11 primary PCR amplification with a concomitant negative 190–200 bp nested GP6/MY11 PCR result, a supplementary long nested PCR using the GP5/MY09 primers was performed to amplify the 380–395 bp segment of DNA downstream of the GP5 primer-binding site. Since the latter long nested PCR is not routinely performed on all samples, this procedure may miss some MY09/MY11 PCR negative cases which can be demonstrated in a GP5/MY09 nested PCR setting only.

The cohort of 3222 patients used in this study consists of two age groups and includes 2633 cases previously analyzed. According to the data reported in the latter study [30], the prevalence rates of the first 5 most common "high-risk" HPV genotypes are HPV-16 (22.9% in women below 30; 14.8% in older women), HPV-52 (6.4% in women below 30; 10.0% in older women), HPV-18 (6.4% in women below 30; 7.1% in older women); HPV-31 (2.7% in women below 30; 4.1% in older women; and HPV-56 (5.5% in women below 30; 2.7% in older women). Based on our experience, HPV-16 seems to be more prevalent in women below 30, and HPV-52 is more frequently detected in women age 30 or older in this suburban US female population who are under the care of board-certified gynecologists in private practice.

When the consensus or degenerate PCR primers are used to amplify a minute quantity of HPV DNA of more than 100 genotypes with varying degrees of mismatching in DNA sequence at the priming sites in an unpurified cervicovaginal sample digestate, co-amplification of non-HPV DNAs derived from the human genome and from other microbes in the vaginal flora is sometimes unavoidable. Under these conditions, each primer seeks to anneal to its most preferred HPV DNA among numerous nucleotides to initiate the enzymatic primer extension reaction. Therefore, most of the nested PCR products can be used for direct automated DNA sequencing reaction without purification because the competing non-HPV DNAs are poorly replicated or not replicated, and simply diluted out in the nested PCR procedure. The L1 genes of the HPV-6, HPV-16, HPV-18, HPV-31, HPV-33 and HPV-35 apparently provide more preferred templates for the consensus PCR primers than those of other genotypes since their nested PCR amplicons usually present themselves as well-defined bands in the expected position with little concomitant high molecular-weight amplicons in the agarose gel after electrophoresis. However, when the target HPV DNA at the priming sites is less preferred by the primers and is amplified with lower efficiencies, the poorly used primers may bind to other competing non-HPV DNAs, causing nonspecific co-amplification during PCR thermocycling, which may in turn generate base calling difficulties in DNA sequencing. This kind of sequencing failure was demonstrated when the DNA of a putative novel subtype of HPV-87 was amplified by the first nested PCR in the presence of competing non-HPV DNAs. Using a second nested PCR in which the HPV DNA of the first nested PCR products was eluted for re-amplification under identical nested PCR conditions, a homogeneous nested PCR product was then generated for a successful DNA sequencing reaction (Figure 2).

The overlapping peaks caused by mixed HPV infections, in which more than 1 HPV DNA is repeatedly co-amplified by the same nested PCR primers, cannot be eliminated by using a second nested PCR amplicon for DNA sequencing. However, since consensus PCR primers do not amplify DNA from different HPV genotypes with the same degree of efficiency, it is possible that one single HPV DNA template is preferentially amplified during its exponential replication under the nest PCR setting to the exclusion of other concomitant HPV in mixed HPV infections. This preferential DNA amplification might have accounted for the low 8% multiple HPV infections observed in this series.

A 34-base sequence immediately downstream of the GP5+ PCR primer site has been proposed as the general signature sequence to identify all known HPV genotypes [20]. We have found that a 34-base sequence is too short for distinguishing some of the closely related HPV genotypes, in particular for some variants of HPV-16, HPV-31 and HPV-33, which share an identical 34-base DNA sequence in this region (Table 2). The more reliable signature sequence for distinguishing the variants of these three genotypes is located in the region upstream of this segment (Figure 1), including part of or the entire GP5+ priming site. For validation of the typing of some variants, a 50-base sequence is needed as unequivocal evidence. The L1 gene DNA sequences of these 3 genotypes are so close to each other that in the same pool of samples, HPV-16 was found to be the most prevalent genotype by one probe hybridization method and HPV-31 the most prevalent by another [16], suggesting that some target DNA of these two HPV variants may have been interchangeably labeled as one type or the other by different probe hybridization methods.

DNA sequencing not only can accurately determine an HPV genotype. It can also unveil novel HPV subtypes or variants, as demonstrated in the case selected for illustration. Sequence alignment algorithm has confirmed that the GenBank database does not contain an HPV sequence fully matched with the sequence of DNA amplified by the nested PCR in this case (Figure 2, lower tracing). The closest sequence alignment is a 90% identities match with the prototype L1 gene of HPV-87 according to the BLAST analysis, making it marginally qualified as an HPV-87, by genotyping definition [19]. On careful analysis, this 72-base DNA sequence encodes an amino acid sequence **NGICWFNQLFVTVVDTTRSTNFTI**, which is 100% matched with the amino acid sequence of the HPV-87 prototype in this region (GenBank Locus AJ400628, HPV-87 putative L1 protein). As a result, this novel HPV is accepted as a putative subtype of HPV-87. Association of HPV-87, a rare HPV genotype, with CIN3 lesions has been reported by others [40,41]. However, this is not the case here because HPV-52 was the only HPV DNA recovered from the paraffin-embedded tissue excised by a cone biopsy which was performed for a CIN3 lesion with extension into endocervical glands three years earlier. One novel HPV-39 variant with a single nucleotide substitute in this hypervariable segment, resulting in change of an amino acid codon (leucine to valine), was observed in a case of persistent HPV infection with a multifocal CIN1 lesion in a 19-year old woman [30]. These findings indicate that DNA sequence variation in this hypervariable region may, or may not, reflect different amino acid composition of the L1 protein in an HPV genotype.

## Conclusion

Direct automated DNA sequencing is a reliable means for validation of HPV genotyping in a routine clinical microbiology laboratory. However, the use of consensus primers for PCR amplification, the identification of the signature sequence for different genotypes on the electropherogram, and the analysis of the BLAST sequence alignment algorithms for genotyping are some of the challenging issues that the technical staff of the laboratory must become familiar with.

## Competing interests

SHL declares that he is a share holder and the president of HiFi DNA Tech, LLC, the company that developed the low-temperature PCR technology. The other authors declare that they have no competing interests.

## Authors' contributions

SHL conceived the study and participated in acquisition, analysis and interpretation of data and in drafting the manuscript. SP participated in direction of the study and analysis of the data with its potential clinical application. VSV and JSV participated in organization of the study, performing the nested PCR, performing the automated DNA sequencing, analyzing the sequencing data and alignment of the computer-generated DNA sequences with those stored in the GenBank to achieve the final HPV genotyping.

All authors have read and approved the final version of the manuscript.

## Pre-publication history

The pre-publication history for this paper can be accessed here:


